# Multi-step real-time prediction of hard-rock TBM penetration rate combining temporal convolutional network and squeeze-and-excitation block

**DOI:** 10.1038/s41598-024-65351-3

**Published:** 2024-06-21

**Authors:** Long Li, ZaoBao Liu, Xingli Fang, Wenbiao Qi

**Affiliations:** 1 School of Management Science and Engineering, Shandong Technology and Business University, Yantai, 264005 Shandong China; 2 Key Laboratory of Ministry of Education on Safe Mining of Deep Metal Mines, College of Resources and Civil Engineering, Northeastern University, Shenyang, 110819 Liaoning China; 3 Department of Computer Science, North Carolina State University, Raleigh, NC 27695 USA; 4 Water Resource and Hydropower Consultative Company of Jilin Province, Changchun, 130021 Jilin China

**Keywords:** Tunnel boring machine, Penetration rate, Temporal convolutional network, Squeeze-and-excitation, Multi-step prediction, Engineering, Civil engineering

## Abstract

Accurate penetration rate prediction enhances rock-breaking efficiency and reduces disc cutter damage in tunnel boring machine (TBM) construction. However, this process faces significant challenges such as the high uncertainty of ground conditions and the complexity of maintaining optimal TBM operation in long and large tunnels. To address these challenges, we propose TCN-SENet++, a novel hybrid multistep real-time penetration rate prediction model that combines a temporal convolutional network (TCN) and a squeeze-and-excitation (SENet) block for aided tunneling. This study aims to demonstrate the application of TCN-SENet++, as well as other models such as RNN, LSTM, GRU, and TCN, for TBM penetration rate prediction. The model was developed using actual datasets collected from the Yin-Song diversion project. We employ a 30-s time step to predict the future time steps of the penetration rate (1st, 3rd, 5th, 7th, and 9th). The features that influence the penetration rate, such as the cutterhead torque, thrust, and cutterhead power, were considered. A comparative analysis using the mean absolute error and mean squared error revealed that the TCN-SENet++ model outperformed the other models, including RNN, LSTM, GRU, TCN, and TCN-SENet+. In comparison, TCN-SENet++ achieved average MSE reductions of 18%, 6%, 3%, 1%, and 2%, respectively. The TCN-SENet++ model demonstrated fewer errors in the new project, validating its effectiveness and suitability for real-time penetration rate prediction in TBM construction.

## Introduction

Tunnel boring machines (TBMs) are widely used in the construction of railroads, municipal transportation, and mining tunnels owing to their high construction speed and safe operation^1,2^. Statistical data show that in 2019 alone, approximately 30 TBMs were used in different projects in China^3^. A crucial aspect of TBM construction involves pushing the disc cutter on the cutterhead into the tunnel face, which represents the interaction between the machine and the ground. The performance of a TBM is assessed based on its penetration rate, which is the ratio of the excavation distance to the operating time^4^. A high penetration rate enhances the rock-breaking efficiency and accelerates the wear of the disc cutter. However, a low penetration rate leads to reduced construction efficiency. Therefore, the penetration rate during excavation is crucial in determining the project schedule and cost^5–7^. Setting the TBM penetration rate requires meticulous consideration of various parameters, including electrical current, torque, and rock chip characteristics. This process is not only time-consuming but also demands significant attention and expertise from operators, particularly when constructing long and large tunnels, where maintaining optimal operation becomes increasingly complex. Furthermore, the complexity of TBM excavation poses significant risks, including working in environments without natural light, the possibility of falling tunnel walls, exposure to various air pollutants, and the risks of explosion and fire, which can lead to irreparable accidents if not properly managed^8,9^. The high uncertainty in ground conditions and limited space further exacerbate these risks.

Considering the continuous operation of TBMs, leveraging intelligent models to predict penetration rates based on big data gathered during TBM operations has made multi-step prediction of penetration rates possible. The purpose of this paper is to develop a multi-step real-time prediction model for hard-rock TBM penetration rate. The model is designed to address the challenges posed by complex and variable excavation conditions, aiming to enhance construction efficiency, improve safety, and reduce project costs by providing operators with precise and timely information. This research focuses on leveraging intelligent models and big data analytics to ensure informed decision-making and optimal TBM performance in large-scale tunneling projects.

Numerous factors influence the penetration rate, including geological conditions, TBM characteristics, site-specific issues, operator experience, contractor management, and expertise^10^. Among these, changes in the geological conditions are of significant importance for TBM construction^11^. Improper penetration rate settings in hard and fractured rocks can cause mechanical damage to the TBM and severe jamming. TBM tunnels are characterized by long distances, high temperatures, and high humidity, which impose substantial labor demands on operators. During the TBM construction process, the operator must consider the current conditions, rock chip morphology, and TBM vibration frequency. Inexperienced operators may struggle to process information inefficiently or provide timely feedback. Construction data are generated by machines interacting with the geology, and these data are abundant. A vast amount of construction data is generated by the interaction of TBMs with geological conditions, making it abundant and valuable. Combining this extensive historical TBM construction data with deep learning algorithms presents a potential avenue for assisted tunneling. Deep learning, particularly when applied to large datasets such as TBM construction data, can continuously enhance accuracy through self-learning processes. Dynamic real-time prediction has emerged as an effective approach for adjusting the penetration rate parameters in response to changing underground environments. By predicting future time steps based on historical TBM data, rock-breaking efficiency and safety can be improved.

**Figure 1:**
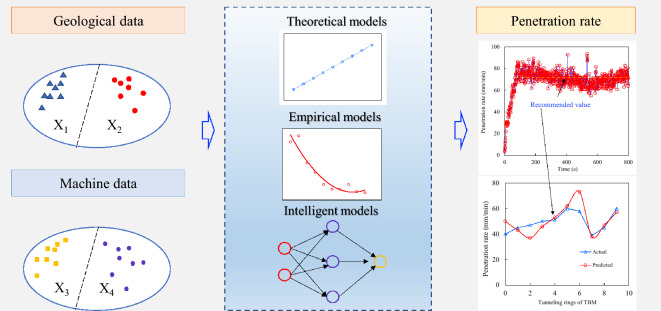
Example of penetration rate prediction for non-time-series models.

Penetration rate prediction models can be classified into two main categories: non-time-series and time-series. Non-time-series models encompass theoretical, empirical, and intelligent models^12^. Construction data are typically collected at a granularity of seconds, and the construction time for each TBM ring exceeds 800 s. For each TBM ring, the penetration rate prediction offers a suggested value, typically representing the average value observed during the stable period of the ring's operation^13^, as shown in Fig. 1.

The classical theoretical model was the Colorado School of Mines (CSM) model^14^. This model considers factors such as tensile strength, tool geometry characteristics, and uniaxial compressive strength. The pressure was uniformly distributed in the contact between the disc cutter and the rock. The theoretical model was built based on ideal conditions, which led to a large error compared with the actual scenario.

The empirical model for predicting the penetration rate, developed by the Norwegian University of Science and Technology (NTNU) through regression analysis^15^, is a notable approach. Moreover, various other scholars have proposed empirical models for predicting penetration rates^13,16–18^. The Brazilian tensile strength, rock brittleness, joint spacing, joint orientation, rock quality designation, and geological strength index are commonly utilized in empirical models. However, some parameters used in the empirical model are more difficult to quantify (e.g., joint conditions)^19^.

Intelligence models with strong nonlinear fitting capabilities are widely used to predict TBM penetration rates, as shown in Table 1. However, existing studies often provide only one reference value for the penetration rate of a TBM ring, and collecting the geological parameters for long tunnels is challenging. Temporal dependencies and limited database sizes were overlooked^20^. Improving penetration rate prediction is crucial for enhancing the efficiency and safety of TBM construction. Addressing these limitations requires incorporating temporal information and innovative methods to better understand the dynamic TBM-geological interactions. Optimizing the process of determining the penetration rates is essential for maximizing construction efficiency and ensuring safe tunneling projects.

**Table 1:** Intelligent prediction model for predicting penetration rate.

Input data		Model	Output	References
Geological parameters	Machine parameters			
UCS, CFF	CT, TF, RPM	ANFIS	PR	^21^
UCS, BI, DPW, *α*	–	ANN	PR	^22^
UCS, DPW, BTS, PSI, *α*	–	FIS	PR	^23^
UCS, DPW, RQD	–	ANN	PR	^24^
UCS, BI, DPW, BTS, *α*	–	PSO	PR	^25^
USC, PSI, DPW, BTS, *α*	–	ANN	PR	^26^
UCS, DPW, BI, *α*	–	FIS	PR	^27^
UCS, BI, BTS, DPW, *α*	TF, CT, SE, CP	SVR	PR	^4^
UCS, DPW, BI, α	–	GWO, DE, HS-BFGS	PR	^28^
UCS, RQD, RMR, GSI, BTS, *J*_*s*_,*Q*, *α*	–	SVR, ANFIS	PR	^29^
UCS, DPW, BI, *α*	–	Bayesian model	PR	^30^
UCS, RQD, BTS, RMR	TF, RPM	PSO-ANN, ICA-ANN	PR	^31^
UCS, DPW, BI, *α*	–	SVR	PR	^32^
UCS, RQD, BTS, WZ, RMR	TF, RPM	GEP	PR	^33^
UCS, RMR, BTS, RQD, WZ	TF, RPM	DNN	PR	^34^
UCS, RQD, GSI, BTS, *J*_*s*_, *α*, etc.	TF, CP, RPM	SVM, ANN	PR	^35^
UCS, BTS, PSI, *α*	–	Monte Carlo-BP neural network	PR	^36^
UCS, RQD, BTS, RMR	TF, RPM	WOA- GEP	PR	^37^
UCS, RQD, RMR, BTS, RMW	TF, RPM	XGB	PR	^38^

UCS: uniaxial compressive strength, CFF: core fracture frequency, B.I.: rock brittleness, DPW: distance between the planes of weakness, *α*: the alpha angle between plane of weakness and TBM driven direction, BTS: Brazilian tensile strength, PSI: peak slope index, *J*_*s*_: joint spacing, *J*_*c*_: joint condition, RQD: rock quality designation, RMR: rock mass rating, GSI: geological strength index, *Q*: quality system, W.Z.: weathered zone, RMW: rock mass weathering, CT: cutterhead torque, T.F.: thrust force, RPM: revolutions per minute, S.E.: specific energy, CP: cutterhead power, ANFIS: adaptive neuro-fuzzy inference system, ANN: artificial neural network, PSO: particle swarm optimization, SVR: support vector regression, GWO: grey wolf optimizer, DE: differential evolution, HS-BFGS: hybrid harmony search, GEP: gene expression programming, DNN: deep neural network, WOA: whale optimization algorithm, XGB: Extreme gradient boosting.

**Figure 2:**
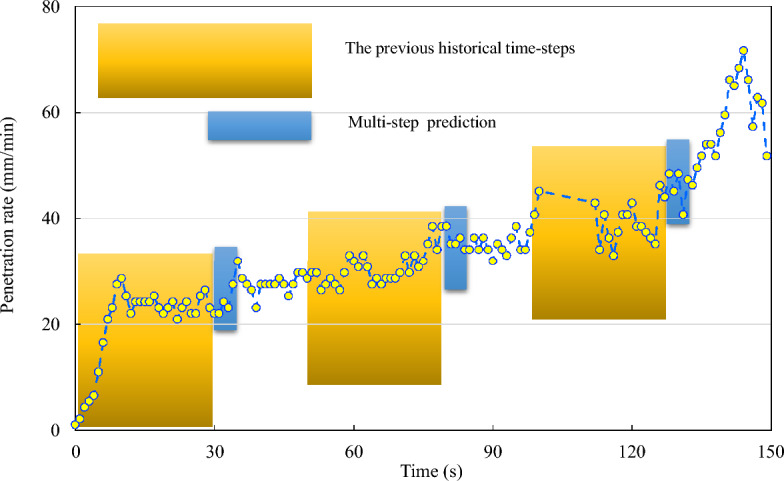
Schematic diagram of multi-step real-time prediction of penetration rate.

Real-time penetration rate predictions enable operators to respond swiftly to complex geological conditions while constructing dynamic TBM tunnels^39,40^. Implementing a big data platform allows for the comprehensive collection of TBM construction data, providing the foundation for real-time penetration rate prediction^41^. The real-time prediction concept relies on time-series correlations in the TBM construction data to forecast future time steps, encompassing both single and multistep predictions (illustrated in Fig. 2). Multistep prediction is particularly challenging because errors can occur over each prediction step. In a multistep prediction scenario, the output of one prediction step becomes the input for the next, making it crucial to maintain high accuracy at each step to avoid a cascading effect of errors. Moreover, the TBM operating environment is highly dynamic and subject to sudden changes in geological conditions that are difficult to capture and predict accurately using conventional models. This dynamic nature requires models that are highly adaptive and robust to unexpected changes. Another significant challenge is the requirement for real-time processing. TBM construction continuously generates vast amounts of data, requiring models that can process and predict penetration rates in real-time without significant lag. This necessitates the use of high-performance computing resources and optimized algorithms to ensure timely and accurate predictions.

This study predicts the future time steps of the penetration rate for every 30-s time step. Currently, multistep real-time penetration rate prediction is applied to Earth pressure balance (EPB) TBMs, which are typically used for soft rock excavation^20^. As EPBs differ significantly from conventional TBMs in terms of balance and support systems, further research is necessary to adapt and optimize the prediction methods for traditional TBM construction^42^. Traditional machine learning models, such as the Support vector machine, random forest, artificial neural network, and Bayesian, are not well suited for handling time-series problems^20,43^. Consequently, deep learning-based time-series forecasting models, such as long short-term memory (LSTM), have been employed for real-time penetration rate prediction^20,39^. LSTM is an improvement over recurrent neural networks (RNNs) in addressing issues such as gradient vanishing and explosion when handling long time-series data, making model optimization challenging. The LSTM network introduces cell states to determine whether information is retained or discarded^44^. Additionally, a gated recurrent unit (GRU) network^45^, based on LSTM has been proposed. However, the initial training errors of the RNN, LSTM, and GRU can be high, and their prediction performance still has certain limitations, particularly when dealing with long time-series data. Further research is required to enhance the prediction capabilities of these models, particularly when handling complex and long-term time-series data encountered during TBM construction.

After the successful application of convolutional neural networks (CNNs) in machine translation, a temporal convolutional neural network (TCN) was introduced after the successful application of convolutional neural networks (CNNs) in machine translation. Initially, CNNs were unsuitable for time-series problems. However, the TCN overcomes this limitation using a one-dimensional convolution and combining causal and dilated convolutions to process time-series data^46,47^. In addition, the TCN inherits the parallel computing capabilities of CNNs. Research has demonstrated that TCNs outperformed RNNs, such as LSTM and GRU networks, in various long time-series tasks^48,49^. Therefore, in this study, a TCN was used for real-time penetration-rate prediction.

However, a limitation of the TCN lies in its uniform weighting of different feature channels, which restricts the performance of the model. It is essential to quantify the disparities between various feature channels in the TCN. To address this issue, a squeeze-and-excitation (SENet) block was introduced. The SENet block considers the weights between different feature channels and obtains these weights through self-learning, allowing it to enhance feature channels relevant to the prediction target^50^. Unlike the introduction of a new spatial dimension, the SENet block considers a sequence of operations and minimizes the loss function to acquire the appropriate weights. In this manner, the SENet block improves the model's ability to emphasize essential features and optimize prediction performance.

This study introduces the TCN-SENet++ deep learning model for the automatic quantification and real-time prediction of penetration rates in TBM construction. The model can automatically learn the laws pertaining to the TBM construction of big data, thereby assisting the driving process. The contributions of this paper are as follows: (1) we improved the SENet block to effectively handle one-dimensional time-series data, enhancing its suitability for TBM construction applications, (2) we proposed a novel hybrid model structure that combines TCN with the enhanced SENet block, and we constructed a residual structure using the SENet block to deepen the model and improve its performance, (3) we conducted a comparative study with classical models such as RNN, LSTM, and GRU, exploring the impact of multi-feature inputs and different time steps on the model's performance, and (4) we validated the generalization capability of the proposed model using a new engineering project, demonstrating its practical utility. These contributions highlight the potential of the TCN-SENet++ model to enhance penetration rate prediction and its practical applicability in TBM construction.

## Methodology

### Temporal convolutional network

Bai Shaojie first proposed TCN in 2018^48^, mainly dealing with the time series forecasting problem. Based on the following principles: (1) the network can generate an output sequence of the same length as the input sequence, (2) future information will not leak to the past, and the TCN adopts a one-dimensional fully convolutional network structure and adds causal convolutions. Next, we introduce the structure of the TCN, which includes causal convolution, dilated convolution, and residual connection.

#### Causal convolutions

**Figure 3:**
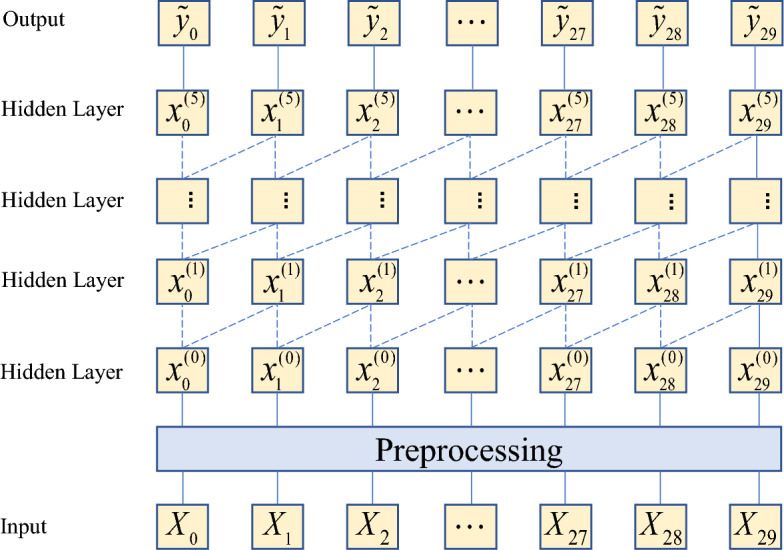
Visualization of causal convolutional layers.

When the model has multiple input features, we define ${\mathbf{X}} = [{\varvec{X}}^{(1)} ,{\varvec{X}}^{(2)} , \ldots ,{\varvec{X}}^{(n)} ] \in {\mathbb{R}}^{n \times T}$, where $T$ is the length of the sequence. Take ${\varvec{X}}^{(i)}$ as an example, ${\varvec{X}}^{(i)} = [X_{1}^{(i)} ,X_{2}^{(i)} , \ldots ,X_{T}^{(i)} ]$. The target sequence is defined as ${\varvec{Y}} = [y_{1} ,y_{2} , \ldots ,y_{T} ] \in {\mathbb{R}}^{T}$. By introducing causal convolutions, TCN ensures that the information at the time $T$ only depends on the previous information, that is, $\tilde{y}_{T + 1}$ is only relevant to ${\varvec{X}}_{1} ,{\varvec{X}}_{2} , \ldots {\varvec{X}}_{T}$, as shown in Fig. 3. For multi-step penetration rate prediction, assuming that the predicted time step is $\lambda$, the formula can be expressed as:

1 $$\tilde{y}_{T + 1} ,\tilde{y}_{T + 2} , \ldots ,\tilde{y}_{T + \lambda } = M({\varvec{X}}_{1} ,{\varvec{X}}_{2} , \ldots {\varvec{X}}_{T} ,{\varvec{Y}})$$

where $M( \cdot )$ is the nonlinear mapping model.

#### Dilated convolutions

**Figure 4:**
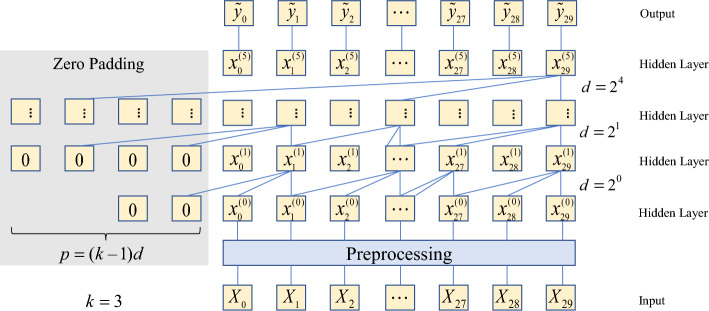
Visualization of dilated convolutional layers.

Increasing the number of causal convolution layers increases the receptive field of convolution in long-term memory. However, this can increase the complexity of the model, leading to a potentially overwhelming computational overhead. Therefore, a dilated convolution was introduced into the TCN to solve the above-mentioned problems, as shown in Fig. 4. For one-dimensional sequence data ${\varvec{X}} = (X_{1} ,X_{2} , \ldots ,X_{T} )$ and filter $F = (f_{0} ,f_{1} , \ldots f_{k - 1} )$, the dilated convolutions of the sequence elements $e$ are as follows:

2 $$F(e) = \sum\limits_{i = 0}^{k - 1} {f(i) \cdot X_{e - d \cdot i} }$$

where $F( \cdot )$ is dilated convolutions operation, $k$ is filter size, $d$ is dilation factor.

The calculation formula for the receptive field is $(k - 1) \, d$, which indicates that a more prominent dilation factor or increased filter size can increase the receptive field. With the filter size $k$ in the convolutional layer fixed, the dilation factor increased exponentially as the depth of the network increased ($i$: $d = 2^{i}$). Therefore, even when the number of convolutional layers was small, the network could obtain a large receptive field.

#### Residual connections

In general, the more convolutional layers, the more features are extracted. Although regularization or dropout can alleviate this issue, convolutional networks are prone to performance degeneracy problems as the number of layers increases, potentially leading to reduced accuracy. He et al.^51^ proposed a residual structure in 2016. The residual structure can realize a cross-layer information transfer. Increasing the depth of a network can effectively resolve the problem of network performance degeneracy. The formula used is as follows:

3 $$o = {\text{Activation}}({\mathbf{X}} + M({\mathbf{X}}))$$

where Activation represents the activation function, ${\mathbf{X}}$ is input date.

**Figure 5:**
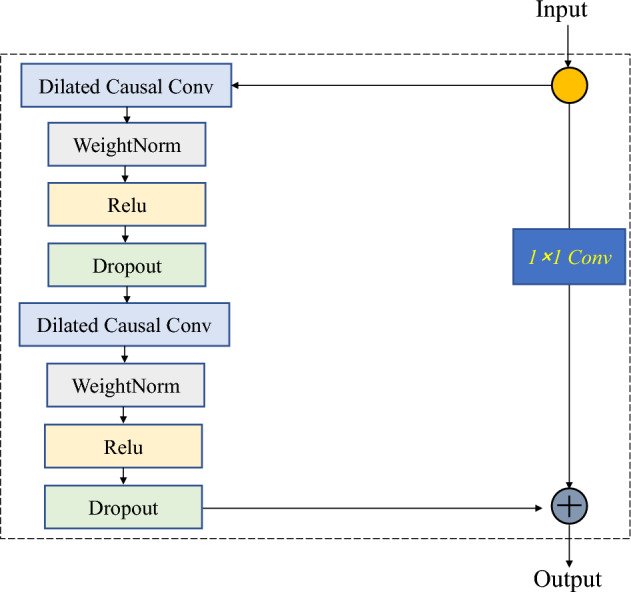
Residual block of TCN.

A TCN is formed by stacking the residual modules, which consist of two layers of causal and detailed convolutions, as shown in Fig. 5. A modified linear unit (rectified linear unit (ReLU)) was used to improve the nonlinear relationship between the convolutional layers. Furthermore, to mitigate the risk of overfitting, dropout was incorporated during the network training phase, in which some neurons were randomly discarded^52^.

### Squeeze-and-excitation block

A CNN employs convolution kernels to extract deep features. To enhance the prediction performance, various methods, such as multi-spatial feature extraction, have been introduced, such as the inception network. In addition to focusing on the network structure, the SENet block considers the interdependence among the input feature channels. During the training process, the network derives weights between different feature channels. These weights help adjust the importance assigned to various feature channels. Upon passing through the TCN, the input features in this study were transformed into a three-dimensional representation, consisting of batch size, channel, and sequence length. Without considering the batch size, the feature channel is defined as ${\mathbf{U}} = [{\varvec{u}}_{1} ,{\varvec{u}}_{2} , \ldots {\varvec{u}}_{C} ] \in {\mathbb{R}}^{T \times C}$, that is, $C$ feature channel of length $T$. As shown below, the SENet block was improved to make it suitable for the multistep real-time prediction of the penetration rate.

#### Squeeze

Squeezing, excitation, and scaling are the three main operations of the SENet block. First, the squeeze operation was analyzed. The TCN outputs multiple feature channels after computing multiple convolutional layers. Global average pooling can quickly and easily calculate the global receptive field of the feature channels. For sequential data, each feature channel is compressed using global average pooling to obtain a scalar that constitutes ${\varvec{z}} = [z_{1} ,z_{2} , \ldots z_{C} ] \in {\mathbb{R}}^{C}$. Thus, all the feature channels form a real-number sequence. The real number of each feature channel was calculated as follows:

4 $$z_{C} = {\mathbf{F}}_{sq} ({\varvec{u}}_{C} ) = \frac{1}{T}\sum\limits_{i = 1}^{T} {u_{C} (i)}$$

where ${\mathbf{F}}_{sq} ( \cdot )$ is the squeeze operation, ${\varvec{u}}_{C}$ represents each feature channel, and $T$ is the feature channel length.

#### Excitation

**Figure 6:**
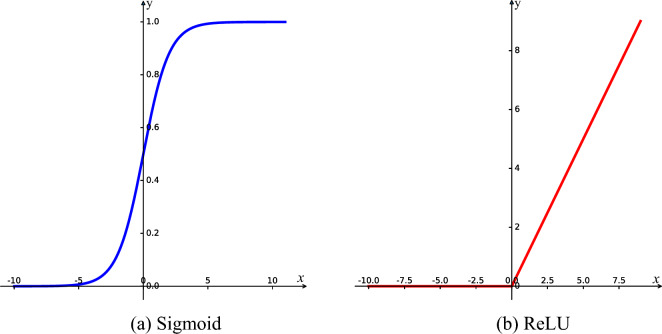
Activation functions.

The global description features of the feature channels are obtained based on the squeeze operation. Therefore, the correlation between feature channels is established by excitation, such that different feature channels obtain different weights ${\varvec{s}}$. This process is calculated using a two-layer fully connected layer, and the formula is as follows:

5 $${\varvec{s}} = {\mathbf{F}}_{ex} ({\varvec{z}},{\mathbf{W}}) = \sigma ((g({\varvec{z}},{\mathbf{W}})) = \sigma ({\mathbf{W}}_{2} \delta ({\mathbf{W}}_{1} {\varvec{z}}))$$

where ${\varvec{s}} = [s_{1} ,s_{2} , \ldots ,s_{C} ] \in {\mathbb{R}}^{C}$, ${\mathbf{W}}_{1} \in {\mathbb{R}}^{{\frac{C}{r} \times C}}$, ${\mathbf{W}}_{2} \in {\mathbb{R}}^{{C \times \frac{C}{r}}}$, $\sigma$ denotes sigmoid activation functions, $\delta$ is the ReLU activation function, $\sigma (x) = \frac{1}{{1 + e^{ - x} }}$, and $\delta (x) = \max (0,x)$. The sigmoid and ReLU activation functions are shown in Fig. 6. The first fully connected layer is mainly used to reduce the dimensionality and thus the calculation, and to increase the nonlinear expression of the network through the ReLU activation function. Conversely, the second fully connected layer increased the dimensionality. Based on the fully connected layer, the model can be adaptively adjusted to ${\mathbf{W}}_{1}$ and ${\mathbf{W}}_{2}$ according to the input features, where $r = 16$ is obtained based on the experiments. The process of first reducing the dimensionality and then increasing it can reduce the complexity of the model and improve its generalization ability. The weight obtained was limited to the range of 0–1 using a sigmoid activation function.

#### Scale

The weights between the feature channels were directly added to the earlier features after calculation. This process was performed using dot-product operations. The formula used is as follows:

6 $$\tilde{\user2{X}}_{C} = {\mathbf{F}}_{scale} ({\varvec{u}}_{C} ,s_{C} ) = s_{C} {\varvec{u}}_{C}$$

where ${\varvec{u}}_{C} \in {\mathbb{R}}^{T}$, $s_{C}$ is scalar, ${\tilde{\mathbf{X}}} = [\tilde{\user2{X}}_{1} ,\tilde{\user2{X}}_{2} , \ldots ,\tilde{\user2{X}}_{C} ]$. By limiting the weight to the range of 0–1, effective feature channels can be enhanced while ineffective feature channels can be suppressed.

### Structure of the real-time penetration rate prediction model

**Figure 7:**
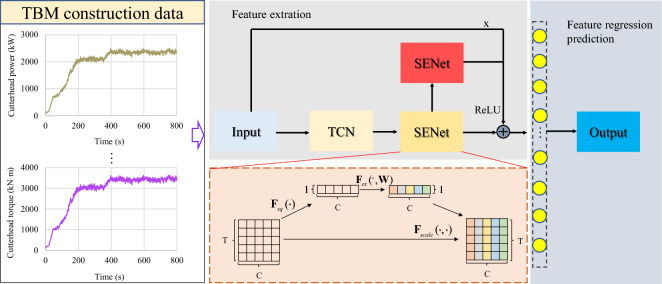
The structure of the TCN-SENet++ model.

A new network structure was proposed by combining the advantages of the TCN and SENet blocks, as shown in Fig. 7. Initially, the key information in the input feature is extracted based on the TCN, utilizing causal and dilated convolutions. The effectiveness of TCN's feature channels is then enhanced using the SENet block, resulting in the TCN-SENet+ model. The SENet block was adapted to process the TBM construction data, and different weights were assigned to the feature channels in the TCN. Weight calculation in the SENet block involves three processes: squeezing, excitation, and scaling. In addition, to further aggregate information from different channels and prevent the degradation of network performance, another SENet block was added after the first SENet block output, forming a residual structure called the TCN-SENet++ model. Finally, a fully connected layer was added to achieve a multistep penetration rate output.

## Project case and feature selection

### Project background and datasets

**Figure 8:**
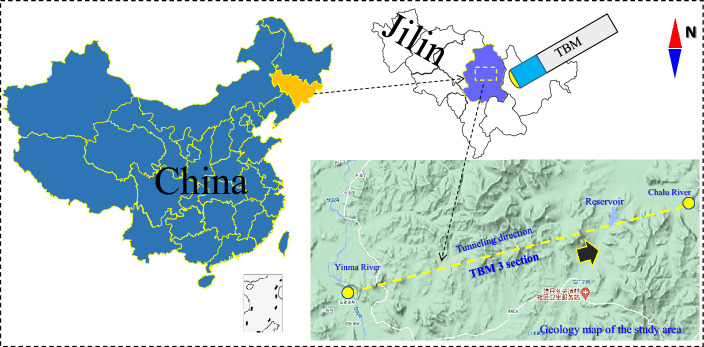
Diagram of Yin-Song diversion project.

The dataset used in this study was obtained from the TBM3 construction section of the Jilin Yin-Song diversion project, as illustrated in Fig. 8. The number of stakes for this project ranged from 71 + 476 to 51 + 705, with a total TBM construction length of 17,488 m. An open-type TBM was employed for the tunnel excavation, and the TBM ring length varied from 0.3 to 1.8 m. The tunnel primarily traverses limestone, granite, tuffaceous sandstone, carbonaceous slate, and diorite. The rock mass class of the tunnel was assessed using the Chinese Hydropower Classification method and mainly falls into classes II, III, IV, and V.

**Figure 9:**
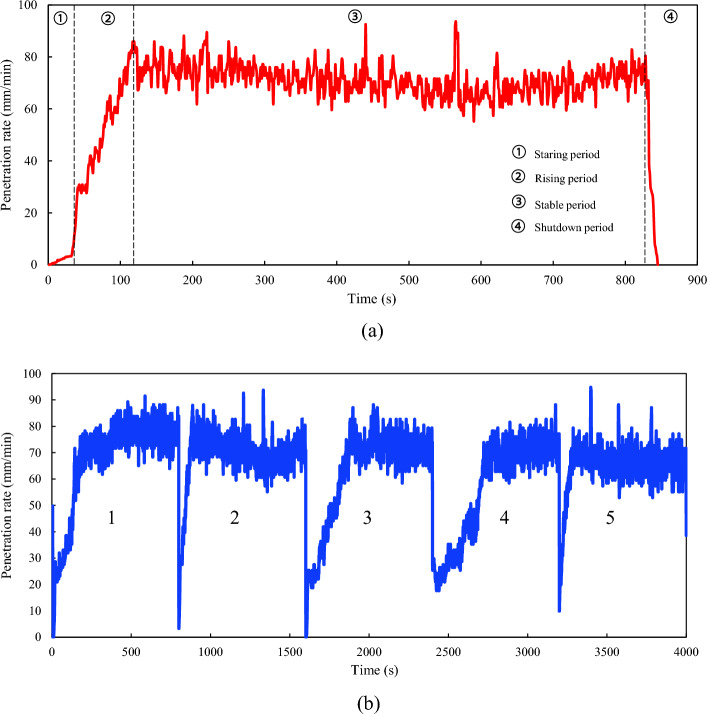
Typical characteristics of TBM parameters: (**a**) four periods of a ring, (**b**) five rings of penetration rate.

The sensors installed in the TBM are capable of real-time data collection and capturing parameters, such as thrust, cutterhead torque, penetration rate, and cutterhead power. The data were stored in a Programmable Logic Controller for further analysis. For this project, the TBM collects 199 parameters per second, and the data collection period spans 728 days. A detailed description of each parameter can be found in the literature reference^41^. The effective ring of the TBM includes starting, rising, stable, and shutdown periods, as shown in Fig. 9a. A specific division algorithm can be found in literature^53^. The rising period determines the parameters of a stable period^41^. However, complex geological conditions caused by TBM vibrations lead to data fluctuations in the stable period of the ring.

Machine learning models typically require inputs of equal length, which means that the length of each ring's data should be the same. To facilitate the calculations, the first 800 s of data for each ring were chosen as an example to investigate the possibility of the real-time prediction of the multistep penetration rate. To ensure computational efficiency, 200 TBM rings were used in this study. The selected data comprise four lithologies, excluding carbonaceous slates, and encompass four classes of rock masses. The penetration rates of the five rings are shown in Fig. 9b.

### Feature selection and data normalized

**Figure 10:**
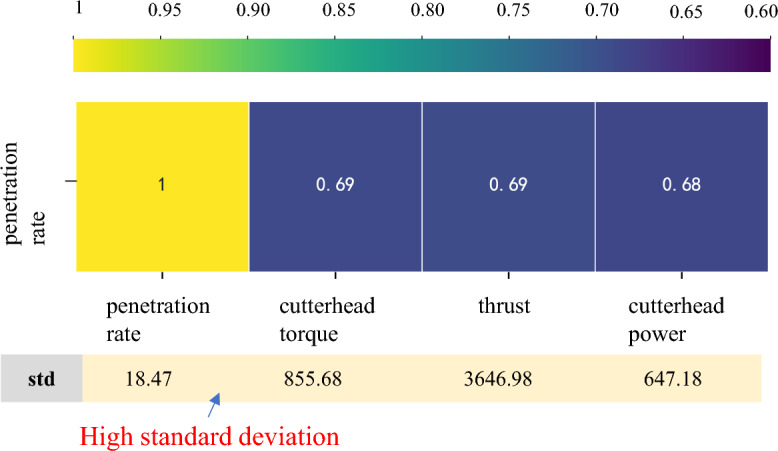
Analysis of the relationship between input features and penetration rate using grey relational grade.

To predict the penetration rate accurately, it is essential to select appropriate input features. In this study, the previous penetration rate is used as an input feature to predict the future penetration rate. Additionally, other parameters, such as the cutterhead torque, thrust, and cutterhead power, are considered, as they also impact the penetration rate. These three features have all been proven to significantly affect the prediction performance of the penetration rate^34,35,39^. Four parameters were chosen as the input features. The grey relational grade (GRG) is used to analyze the relationship between these four input features and the penetration rate. A higher GRG value indicates a more favorable prediction of the penetration rate, suggesting a stronger correlation between the input features and the target variable^54^. GRG values greater than 0.5 indicate a strong relationship between features and predicted targets^55^. As shown in Fig. 10, the GRG values of the input features exceeded 0.5.

**Figure 11:**
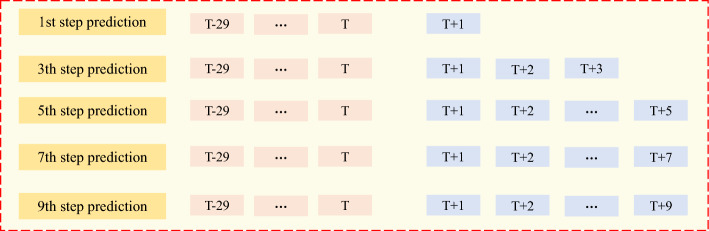
The multi-step prediction schematic.

Li et al.^41^ achieved accurate predictions of TBM parameters during the stable period by using the first 30 s of data from the rising period. Similarly, we aim to conduct a multi-step real-time prediction of the penetration rate using a 30-s time step. In Fig. 11, we illustrate the odd timestep predictions, specifically the 1st step, 3rd step, 5th step, 7th step, and 9th step.

The TBM construction data were preprocessed, as described in Ref.^7^. The scale of the input feature is different, resulting in slow convergence of the neural network loss function. Therefore, it was necessary to normalize the input features^7^. The formula used is as follows:

7 $$X^{\prime} = \frac{{X - \overline{X}}}{\sigma }$$

where $X^{\prime}$ represents the data after normalization, $X$ represents the original TBM data, $\overline{X}$ and $\sigma$ represent the mean and standard deviation of the original data.

## Development of the real-time penetration rate prediction model

Optimizing the hyperparameters of a model is essential for assessing and comparing the performances of different models. The selection of appropriate evaluation criteria is equally important. In this study, classical RNN-based models were selected for comparison with TCN-based models. The optimization range of the hyperparameters was determined based on the characteristics of the established TBM dataset.

Drawing on the optimized hyperparameters for RNN, LSTM, GRU, and TCN from previous scholars' work^48^, the hyperparameters optimized for RNN-based models included the number of hidden layers, number of hidden layer neurons, and learning rate. For the TCN-based models, the optimized hyperparameters include the convolutional kernel size, number of convolutional layers, number of convolutional layer channels, and learning rate. Model training employed the Adam adaptive optimizer, which is popular in time-series predictions^56^.

### Evaluation criteria

Two indicators were introduced into the evaluation model for the regression problem: mean absolute error (MAE), mean square error (MSE) and coefficient of determination (R^2^). The formula used is as follows:

8 $${\text{MAE}} = \frac{1}{m}\sum\limits_{i = 1}^{m} {\left| {y_{i} - \widehat{y}_{i} } \right|}$$

9 $${\text{MSE}} = \frac{1}{m}\sum\limits_{i = 1}^{m} {(y_{i} - \widehat{y}_{i} )^{2} }$$

10 $${\text{R}}^{2} = 1 - \frac{{\sum\nolimits_{i = 1}^{m} {(y_{i} - \widehat{y}_{i} )^{2} } }}{{\sum\nolimits_{i = 1}^{m} {(y_{i} - \overline{y}_{i} )^{2} } }}$$

where m is the number of TBM rings, $y_{i}$ is the actual value at time* i*, and $\hat{y}_{i}$ is the predicted value at time *i*.

### Hyperparameter optimization method

Different hyperparameter combinations, such as the learning rate and number of neurons, can significantly affect the performance of machine learning models. Various algorithms have been proposed to optimize these hyperparameters, including grid and random grid searches. The grid search algorithm is well-suited for relatively fewer combinations of model hyperparameters. It systematically explores all combinations within predefined ranges to determine the optimal values. In contrast, the random search algorithm randomly selects samples from the hyperparameter space. The idea is that the global optimal value can be determined using enough random samples. However, the accuracy of the random search algorithm may have certain limitations. Each optimization algorithm has its characteristics and applicable conditions. Researchers and practitioners often select the most appropriate algorithm based on the complexity of the hyperparameter search space and the available computational resources^57^.

A successive halving method is proposed to overcome the limitations of the model. This involved multiple training and evaluation rounds. In each round, the model was trained using all the hyperparameter combinations, and its performance was evaluated. Poorly performing combinations were eliminated and the process was repeated until only a few potential combinations remained. The model with the best performance was selected based on the remaining combinations. This method efficiently prunes the hyperparameter search space, leading to an optimal hyperparameter selection^58^. The asynchronous successive halving algorithm improves the optimization speed by allowing the concurrent evaluation of the hyperparameter combinations that enter the next round while the current round is ongoing. This reduces the waiting time between rounds and accelerates the optimization process, leading to a better model performance^59^. This strategy can fully utilize the computing resources and significantly improve the computing efficiency. The method was implemented within the Ray Library^60^ using Python, allowing the optimization of deep-learning models based on the PyTorch framework. Consequently, all hyperparameter optimization processes in this study were conducted using Ray as the foundation.

In this study, different hyperparameter combinations, such as the learning rate and number of neurons, were extensively experimented with, as these can significantly affect the performance of machine learning models. Various algorithms, including grid search and random search, were used to optimize these hyperparameters. To overcome the limitations of these traditional methods, we implemented the successive halving method, which involves multiple rounds of training and evaluation. Poorly performing combinations were eliminated in each round until the best-performing model was selected. Additionally, the asynchronous successive halving algorithm was employed to improve optimization speed by allowing concurrent evaluations, thereby reducing waiting time and accelerating the process. All hyperparameter optimization processes were conducted using the Ray Library in Python, enabling efficient optimization of deep-learning models based on the PyTorch framework. Through this comprehensive approach, we systematically experimented with and optimized hyperparameter values, ensuring the robustness and accuracy of our models. Sensitivity analysis showed that parameters such as learning rate and number of neurons significantly influenced the results, highlighting the importance of thorough hyperparameter optimization.

In this study, we thoroughly experimented with various sets of hyperparameter values, including the learning rate and number of neurons, to determine their impact on model performance. By employing the successive halving method, we systematically explored the hyperparameter space. This method allowed us to efficiently prune less promising combinations and focus on those with higher potential, thus optimizing the overall model performance.

### Hyperparameter optimization range

Currently, most related research focuses on RNN-based models, particularly the RNN, LSTM, and GRU models, for TBM parameter prediction. Considering the characteristics of the RNN-based models, we optimized the number of hidden layers, the number of hidden layer neurons, and the learning rate. The specific hyperparameter optimization ranges are listed in Table 2.

**Table 2:** Hyperparameters search ranges of RNN-based models.

Hyperparameters	Tunning range
RNN-based layer unit	[32, 64, 128]
Number of hidden layers	[1–3]
Learning rate	[0.004–0.01]

The TCN-based models were TCN, TCN-SENet+, and TCN-SENet++, in which the convolutional kernel size, number of convolutional layers, number of convolutional layer channels, and learning rate of the models were optimized^48^. For simplicity, the number of neurons in the fully connected layer was set equal to the number of channels in the convolutional layer. Considering the characteristics of the dataset and the number of channels, the dropout layer parameter in the TCN was configured to have a value of 0.2^48^. The hyperparameter optimization range of the TCN-based model is listed in Table 3.

**Table 3:** Hyperparameters search ranges of RNN-based models.

Hyperparameters	Tunning range
Kernel size	[2–6]
Number of convolution layers	[3–6]
Number of channels	[32, 64, 128]
Learning rate	[0.004–0.01]

**Figure 12:**
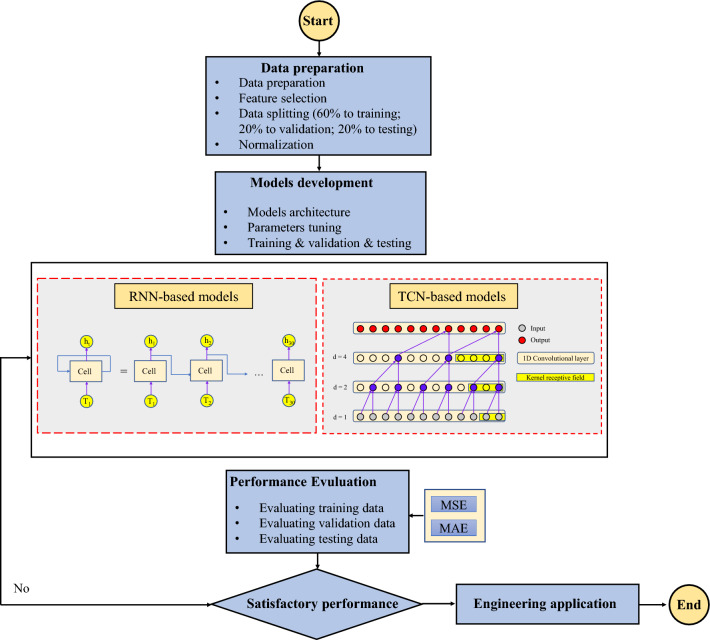
Flowchart of penetration rate prediction model.

The training period of the model was defined as 200 epochs, and the MSE was used. To strike a balance between the local optimum and the computer performance, the batch size was set to 100. To mitigate model overfitting, an early stopping method^61^ was employed, which terminated the training if the loss function did not decrease after 20 epochs. The modeling process is illustrated in Fig. 12. The dataset was divided into 60%, 20%, and 20% for training, validation, and test sets, respectively.

Research has indicated that the batch size and dropout hyperparameters are primarily used to address overfitting issues during model training. Because there were no overfitting problems in our model training, we set these parameters to 100 and 0.2, respectively. In addition, adaptive optimizers, such as Adam, perform well without extensive tuning^62^. Therefore, this study did not conduct an in-depth optimization of the batch size and dropout hyperparameters.

## Prediction results of different models

Thus, the proposed TCN-SENet++ model is suitable for time-series forecasting. It exhibited good generalization performance across the training, validation, and test sets. A comparison with popular RNN-based models was conducted to analyze the applicability of different models. The results were analyzed as follows. The PyTorch library with a Jupyter Notebook was used in this study. A computer with a 3.60 GHz Intel Core i9-9900k, 64 GB of memory, and an NVIDIA GeForce GTX 2080ti graphics card was used for all the tests.

The performance of the model was evaluated using the training, validation, and test sets. As presented in Table 4, the TCN performs best in the 1st, 7th, and 9th steps, whereas TCN-SENet++ excels in the 3rd and 5th steps in the training set. Among the RNN-based models, RNN performed the worst and GRU performed the best. Overall, TCN-SENet++ achieved the best performance, with the lowest average MSE and MAE values.

**Table 4:** Comparison of the different models on the training set for multiple features.

Model	Evaluating indicator	1-step	3-step	5-step	7-step	9-step	Average
RNN	MSE	22.857	19.439	22.232	23.958	32.882	24.274
	MAE	3.670	3.330	3.533	3.665	4.295	3.699
LSTM	MSE	15.327	18.558	20.299	22.072	25.017	20.255
	MAE	2.996	3.257	3.382	3.517	3.740	3.378
GRU	MSE	15.136	18.256	21.696	22.968	24.379	20.487
	MAE	**2.982**	3.231	3.507	3.591	3.700	3.402
TCN	MSE	**14.877**	17.282	20.29	**21.848**	**23.335**	19.526
	MAE	2.957	3.163	3.391	**3.505**	**3.617**	3.327
TCN-SENet+	MSE	15.782	17.92	20.208	22.880	23.710	20.100
	MAE	3.046	3.225	3.386	3.580	3.658	3.379
TCN-SENet++	MSE	15.272	**16.282**	**19.517**	22.202	23.741	**19.403**
	MAE	2.995	**3.078**	**3.334**	3.531	3.641	**3.316**
	R^2^	0.947	0.944	0.930	0.923	0.917	0.932

Significant values are in bold.

As shown in Table 5, TCN-SENet++ demonstrated the best performance in the validation set, followed by TCN. The GRU outperformed both the RNN and LSTM. Notably, the GRU achieved the best performance in 1st step prediction with an MSE of 16.118 and an MAE of 3.034. Furthermore, when the average MSE and MAE of the validation sets were compared, TCN-SENet++ exhibited the best overall performance.

**Table 5:** Comparison of the different models on the validation set for multiple features.

Model	Evaluating indicator	1-step	3-step	5-step	7-step	9-step	Average
RNN	MSE	23.640	21.470	25.123	27.083	34.254	26.314
	MAE	3.662	3.456	3.691	3.809	4.301	3.784
LSTM	MSE	16.900	20.419	24.344	26.607	29.023	23.459
	MAE	3.087	3.358	3.604	3.760	3.916	3.545
GRU	MSE	**16.118**	19.741	23.856	25.843	28.669	22.845
	MAE	**3.034**	3.313	3.620	3.724	3.900	3.518
TCN	MSE	16.567	19.746	23.008	**25.182**	**26.763**	22.253
	MAE	3.069	3.314	3.524	**3.667**	**3.675**	3.450
TCN-SENet+	MSE	16.975	20.400	23.360	25.468	27.481	22.737
	MAE	3.109	3.376	3.558	3.697	3.826	3.513
TCN-SENet++	MSE	16.783	**19.560**	**22.840**	25.199	**26.763**	**22.229**
	MAE	3.094	**3.297**	**3.508**	3.676	3.775	**3.470**
	R^2^	0.941	0.931	0.919	0.911	0.905	0.921

Significant values are in bold.

TCN-SENet++ exhibited a better performance in the training and validation sets. The performance of the model on the test set was compared to evaluate its generalizability, as listed in Table 6. For the 1st step prediction, GRU had the best performance with an MSE and MAE of 17.252 and 3.101, respectively. For the 3rd step prediction, TCN-SENet++ exhibited the best performance, with an MSE and MAE of 20.762 and 3.343, respectively. For the 5th step prediction, TCN-SENet++ had the best performance, with an MSE and MAE of 24.099 and 3.548, respectively. For the 7th step prediction, TCN-SENet++ had the best performance, with an MSE and MAE of 26.793 and 3.704, respectively. For the 9th step prediction, TCN-SENet++ had the best performance, with an MSE and MAE of 28.282 and 3.802, respectively.

**Table 6:** Comparison of the different models on the test set for multiple features.

Model	Evaluating indicator	1-step	3-step	5-step	7-step	9-step	Average
RNN	MSE	24.639	22.821	26.212	28.672	36.703	27.809
	MAE	3.687	3.503	3.713	3.846	4.412	3.832
LSTM	MSE	17.977	21.884	25.686	28.593	31.023	25.033
	MAE	3.159	3.42	3.646	3.808	3.952	3.597
GRU	MSE	**17.252**	21.366	25.158	27.508	30.53	24.363
	MAE	**3.101**	3.385	3.641	3.778	3.926	3.566
TCN	MSE	17.319	21.017	24.615	26.848	29.123	23.784
	MAE	3.114	3.372	3.570	3.698	3.834	3.518
TCN-SENet+	MSE	17.948	21.417	24.813	26.812	29.269	24.052
	MAE	3.180	3.417	3.604	3.734	3.866	3.560
TCN-SENet++	MSE	17.838	**20.762**	**24.099**	**26.793**	**28.282**	**23.555**
	MAE	3.156	**3.343**	**3.548**	**3.704**	**3.802**	**3.511**
	R^2^	0.930	0.918	0.905	0.893	0.887	0.906

Significant values are in bold.

TCN-SENet++ exhibited superior performance on both the training and validation sets. To further evaluate its generalization ability, the performance of the model was compared with that of the test set, as listed in Table 6. For the 1st step prediction, the GRU achieved the best performance, with an MSE of 17.252 and MAE of 3.101. For the 3rd step prediction, TCN-SENet++ performed the best with an MSE of 20.762 and an MAE of 3.343. For the 5th step prediction, TCN-SENet++ outperformed the other models, with an MSE of 24.099 and an MAE of 3.548. For the 7th step prediction, TCN-SENet++ demonstrated the best performance, with an MSE of 26.793 and an MAE of 3.704. For the 9th step prediction, TCN-SENet++ continued to excel with an MSE of 28.282 and an MAE of 3.802. These results highlighted the strong generalizability of TCN-SENet++ for time-series forecasting across multiple steps.

Among the RNN-based models, the GRU achieved the best performance, followed by the LSTM. However, the RNN exhibited the worst generalization ability, particularly for the 9th step prediction, with an MSE of 36.703 and MAE of 4.412. Compared with TCN-SENet+, the TCN showed a better overall performance. The inclusion of the SENet block increased the computational complexity of the model, leading to model degradation. By contrast, TCN-SENet++ overcomes this issue by incorporating a residual structure, which enhances the generalization ability of the model and prevents degradation.

The performance of the model decreases as the prediction time step increases. In terms of the MSE and MAE, the RNN showed the worst performance, followed by the LSTM. The GRU outperformed the RNN and LSTM. The performance of TCN-SENet++ was better than that of TCN and TCN-SENet+, with MSE and MAE of 23.555 and 3.511, respectively. Therefore, the proposed model exhibits optimal performance.

Subsequently, we utilized the R^2^ to further evaluate the predictive capabilities of the TCN-SENet++ model across the training, validation, and testing datasets. As depicted in Tables 4, 5, and 6, an increase in the prediction step length correlates with a decrease in R^2^, signifying a diminished model performance. This observation aligns with insights obtained from the MSE and MAE analyses. In the testing dataset, the model achieved an average R^2^ of 0.906, further demonstrating the effectiveness of multi-step forecasting of the penetration rate in practical engineering applications.

**Figure 13:**
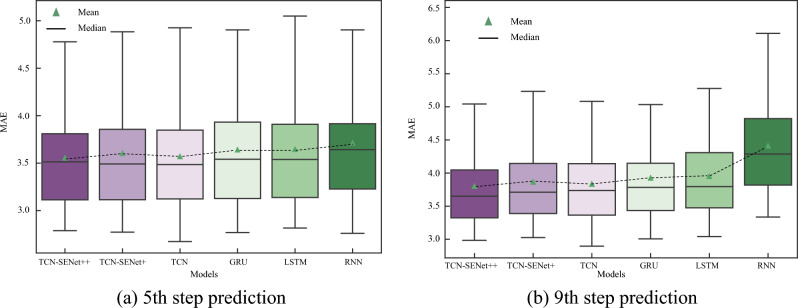
Boxplot of MAE distribution: (**a**) 5th step prediction, (**b**) 9th step prediction.

The proposed TCN-SENet++ model exhibited optimal performance in the test set. For 5th step and 9th step predictions, the results demonstrate the effectiveness of TCN-SENet++. To compare the performance of the different models in predicting the penetration rate, a boxplot was used to visualize the MAE distribution range of each model for each ring in the test set (as shown in Fig. 13). A boxplot was used to display the data distribution characteristics. When comparing TCN-based models to RNN-based models, the latter tended to have a larger error range. Introducing a fully connected layer in the SENet block increases computational complexity and can slightly affect the performance of the TCN, leading to a broader MAE distribution range. Nevertheless, the proposed TCN-SENet++ model demonstrated superior performance with a smaller range of MAE distributions, highlighting its ability to make accurate predictions for each ring in the test set.

**Figure 14:**
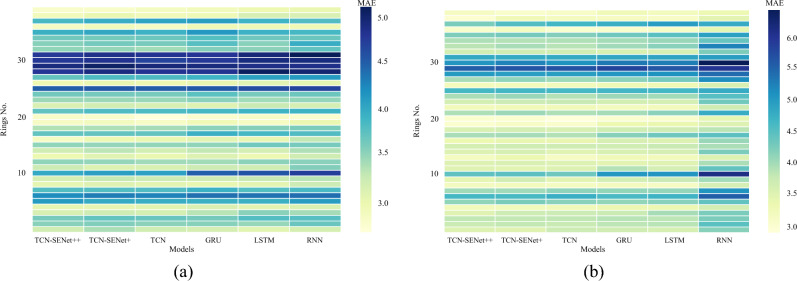
Heatmap of MAE distribution: (**a**) 5th step prediction, (**b**) 9th step prediction.

Figure 14 illustrates the MAE for each ring in the 5th and 9th step predictions steps. The color intensity of the rectangle corresponds to the MAE value, with darker shades indicating larger MAE values. Notably, all models exhibited high MAE values for rings 25, 28–31. Further analysis revealed that rings No. 28–29 had high standard deviations of the penetration rates. Consequently, the performance of all the models deteriorated in these rings. The poor performance in rings with a high standard deviation indicates that the models struggle to predict the penetration rates accurately when dealing with such volatile and uncertain data.

**Figure 15:**
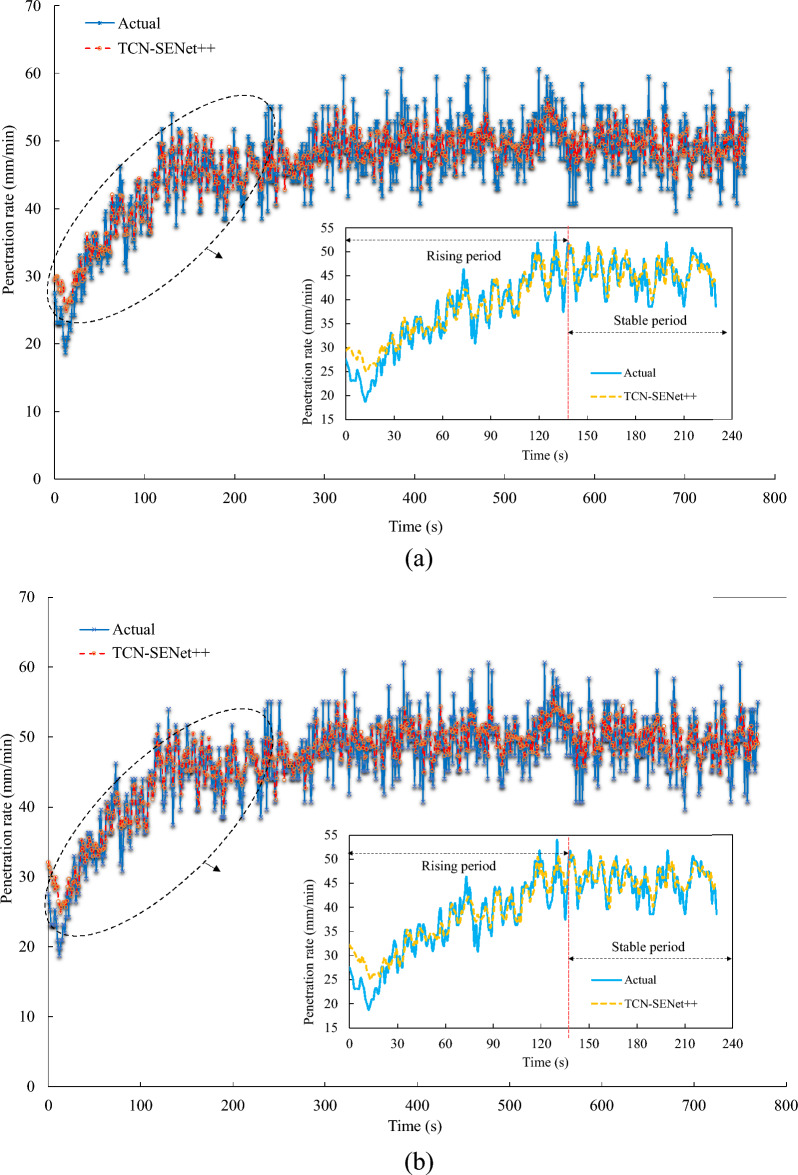
Comparison of actual and predicted penetration rate curves of TCN-SENet++: (**a**) 5th step prediction, (**b**) 9th step prediction.

**Figure 16:**
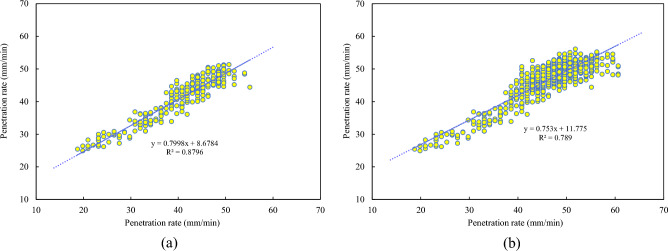
Scatter plots of 5th step prediction of actual and predicted values on the test set: (**a**) the first 240 s of ring No. 40, (**b**) the whole ring No. 40.

In this study, ring 40 from the test set served as an example to visualize the prediction results of the developed TCN-SENet++ model. As shown in Fig. 15, the predicted values aligned closely with the actual values of the penetration rate. This demonstration validates the feasibility of the multistep prediction based on historical data. To further assess the applicability of the model, the ${R}^{2}$ (coefficient of determination) for the first 240 s of ring No. 40 and the entire ring were analyzed, as illustrated in Figs. 15 and 16. The fit coefficients of the rising period were greater than 0.87, indicating that the model effectively captured the data patterns during this phase. This capability is beneficial for operators in accurately setting the penetration rate. However, during the stable period with high penetration rates, the TBM vibration frequency increases, leading to greater data dispersion. Consequently, the fit coefficients of the predicted results decreased during this phase. Nevertheless, the model remains valuable for predicting the penetration rate during the rising period and assisting operators in making informed decisions.

Although the proposed model captured the overall trend of the penetration rate sequence, the prediction results showed opposite trends at certain time points (e.g., the trends around time of 25 s, 50 s or 80 s). This issue can have negative impacts on practical engineering applications. To address this problem, increasing the number of tunneling rings to expand the dataset is currently an effective solution, along with improving the computational performance of the GPU. When such issues arise in practical applications, shortening the prediction steps as much as possible can reduce prediction errors.

## Discussion

The discussion section contains three sections. First, only the impact of historical data on the penetration rate for future time-step predictions was analyzed. Second, the training time, loss function convergence of the different models, and effect of different historical time steps on the prediction results were analyzed. Finally, the proposed model was migrated to another new project to explore its adaptability and embed the model into a TBM intelligent construction system.

### Single feature multi-step prediction penetration rate analysis

Table 7 presents the test set results used to evaluate the generalization ability of the model. The average MSE and MAE values for this set were 24.234 and 3.576, respectively. When comparing these results with those in Table 6, it is evident that using only historical penetration rate data to predict future time steps leads to lower accuracy, with an MSE of 29.672 and an MAE of 3.893 in the 9th step. Conversely, considering multiple features, such as cutterhead torque, thrust, and cutterhead power, improved performance, with an MSE of 28.282 and an MAE of 3.802.

**Table 7:** Comparison of the different models on the test set for a single feature.

Model	Evaluating indicator	1-step	3-step	5-step	7-step	9-step	Average
RNN	MSE	25.904	28.558	26.773	31.181	37.473	29.978
	MAE	3.856	3.950	3.744	3.954	4.442	3.989
LSTM	MSE	18.200	23.086	26.421	29.196	31.845	25.750
	MAE	3.180	3.513	3.700	3.852	3.987	3.646
GRU	MSE	18.047	22.357	25.807	28.347	31.32	25.176
	MAE	3.157	3.475	3.670	3.848	3.960	3.622
TCN	MSE	18.032	21.945	25.592	28.306	29.694	24.714
	MAE	3.189	3.450	3.653	3.830	**3.893**	3.603
TCN-SENet+	MSE	17.994	22.144	25.503	27.789	30.233	24.733
	MAE	3.187	3.478	3.667	3.798	3.926	3.611
TCN-SENet++	MSE	**17.849**	**21.509**	**24.6**	**27.539**	**29.672**	**24.234**
	MAE	**3.172**	**3.421**	**3.615**	**3.782**	**3.893**	**3.576**

Significant values are in bold.

These findings highlight the importance of incorporating multiple features in the prediction model. Although historical penetration rate data alone can provide a baseline for predictions, the inclusion of additional parameters significantly enhances the model's accuracy. This suggests that while the assumption that single-feature models can predict penetration rates is valid, a multi-feature approach is more effective. Therefore, it is crucial to include cutterhead torque, thrust, and power in the input features to achieve more reliable and accurate predictions.

### Time consumption time, loss function and time step analysis

**Figure 17:**
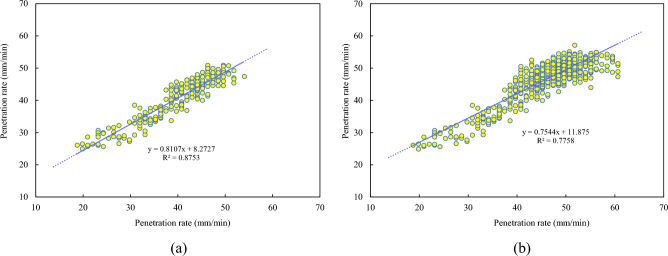
Scatter plots of 9th step prediction of actual and predicted values on the test set: (**a**) the first 240 s of ring No. 40, (**b**) the whole ring No. 40.

The average training times of the different models were compared to assess their applicability. As depicted in Fig. 17, the RNN had the shortest average training time of 316.718 s, followed by the GRU with a training time of 363.162 s. RNN-based models use sequence data as input and utilize the chain structure of the network to incorporate the memory function, enabling them to capture the relationships within the TBM construction data. However, RNN exhibits poor performance in long-distance time-series prediction problems, such as the 9th-step prediction. LSTM addresses this limitation by introducing three gating units (forget, input, and output gates) to ensure training stability and improve prediction performance. Consequently, the training time for an LSTM is longer than that for an RNN. The GRU, derived from the LSTM, simplifies the gating mechanism by employing only two gating units: the update gate and the reset gate. Therefore, the GRU's training time is longer than that of the RNN but shorter than that of the LSTM.

**Figure 18:**
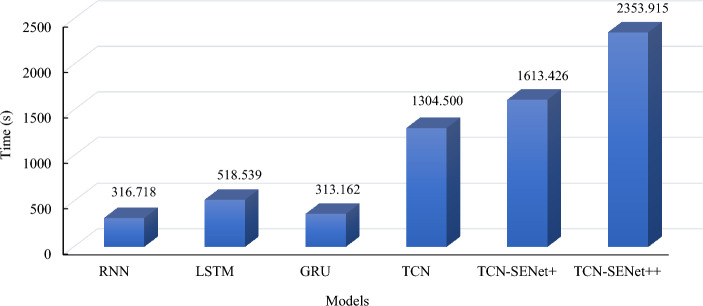
Comparison of training time of different models.

Figure 18 shows that the TCN-based models required much longer training times than the RNN-based models. TCN's increased numbers of layers and channels for capturing long-term historical information contributed to extended training times. TCN-SENet++ exhibited the best performance but also the longest training time, averaging 2353.915 s. Time costs must be considered when using TCN-SENet++ in practical applications.

**Figure 19:**
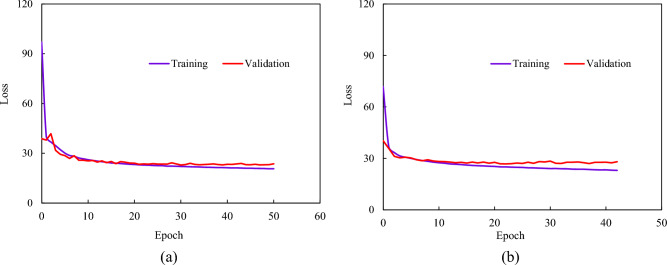
Training and validation loss of TCN-SENet++: (**a**) 5th step prediction training and validation loss, (**b**) 9th step prediction training and validation loss.

The training and validation losses of the 5th and 9th step predictions were analyzed as examples to evaluate the existing overfitting problems in TCN-SENet++. As shown in Fig. 19, the validation loss is consistent with the training loss, and TCN-SENet++ has no overfitting problems. This also demonstrates the reasonableness of the selected input features.

**Figure 20:**
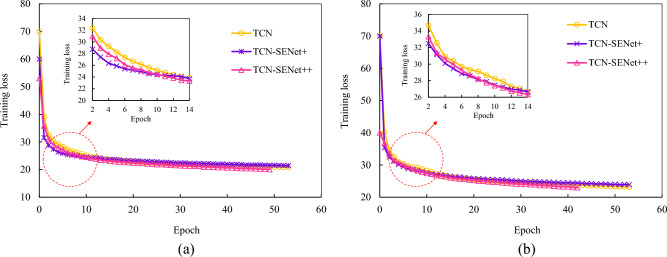
Training loss of TCN-based model: (**a**) 5th step prediction training loss, (**b**) 9th step prediction training loss.

The SENet block minimizes the loss function by calculating the weights between different feature channels. Figure 20 shows that the TCN initially has the largest loss value during early training, whereas TCN-SENet+ experiences an increased error in the later stages owing to the addition of layers. In contrast, TCN-SENet++ effectively prevented overfitting and improved model performance by incorporating a residual structure. Overall, TCN-SENet++ is the most effective model for enhancing performance and preventing degradation.

**Figure 21:**
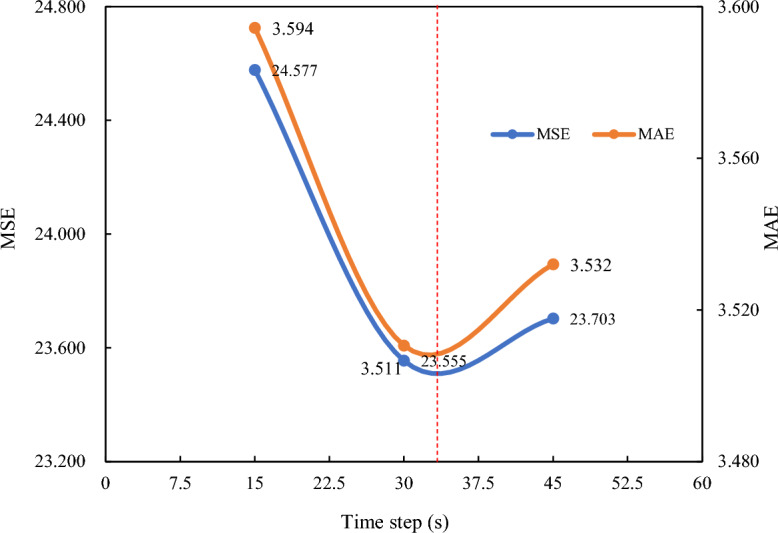
MSE and MAE of different time steps using TCN-SENet++.

Using a 30-s time step to predict future permeability was found to be feasible. However, the impact of different time steps on multistep real-time prediction of the penetration rate requires further clarification. Figure 21 presents the analysis of the MSE and MAE of the TCN-SENet++ model at various time steps (15, 30, and 45-s time steps) using the test set. Compared to the 15-s time step, the MSE was reduced by 4% for the 30-s time step. Additionally, the MSE increases by 0.06% for the 60-s time step compared to the 30-s time step. Notably, the model showed a significant performance improvement for time steps ranging from 15 to 33 s. However, for time steps between 33 and 45 s, the performance improvement was limited. This suggests that modeling historical TBM construction data for future penetration rate prediction becomes challenging when dealing with long time-series data (time steps beyond 33 s).

### Engineering application of the proposed model

**Figure 22:**
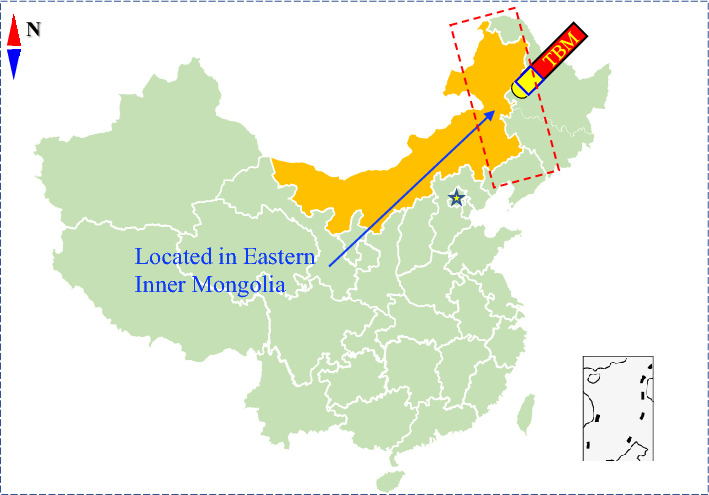
The location of a new project.

As depicted in Fig. 22, a project located in eastern Inner Mongolia, China, served as a crucial testbed to demonstrate the generalization capabilities of the proposed model. Therefore, these three parameters were selected as input features to retrain the model. The same number of rings was selected as the test set for testing, that is 40. The surrounding rock class in the selected data was mainly Class II and the lithology was tuffaceous.

As shown in Table 8, the project evaluated the performances of the six models. It is critical to emphasize that the data in this project were not used as training data, but rather to directly test the performance of the model. The proposed TCN-SENet++ model exemplifies excellent generalization capabilities. Notably, this model not only demonstrated high predictive accuracy but also outperformed other models at various forecasting horizons, specifically in the 1st, 3rd, 5th, 7th, and 9th step predictions. The TCN has the best performance in 1st step prediction, whereas the GRU has the best performance in 3rd step prediction.

**Table 8:** Comparison of the different models in a project.

Model	Evaluating indicator	1-step	3-step	5-step	7-step	9-step	Average
RNN	MSE	20.489	20.085	21.917	21.666	23.719	21.575
	MAE	3.532	3.499	3.645	3.675	3.804	3.631
LSTM	MSE	17.144	16.485	20.274	18.340	22.062	18.681
	MAE	3.206	3.183	3.546	3.343	3.720	3.399
GRU	MSE	14.621	**15.887**	20.163	21.635	23.514	19.164
	MAE	2.992	**3.107**	3.545	3.665	3.852	3.432
TCN	MSE	**13.506**	16.826	19.289	20.187	22.262	18.414
	MAE	**2.876**	3.206	3.437	3.527	3.721	3.353
TCN-SENet+	MSE	14.732	16.745	20.260	19.138	19.347	18.044
	MAE	3.000	3.200	3.521	3.428	3.449	3.319
TCN-SENet++	MSE	14.367	16.599	**16.969**	**17.793**	**18.812**	**16.908**
	MAE	2.956	3.176	**3.229**	**3.312**	**3.414**	**3.217**

Significant values are in bold.

**Figure 23:**
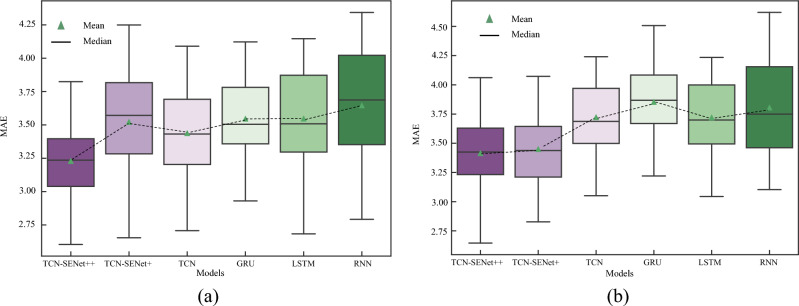
Boxplot of MAE distribution in a new project: (**a**) 5th step prediction, (**b**) 9th step prediction.

Similarly, considering the 5th and 9th step predictions as examples, the prediction results of TCN-SENet++ are shown in Fig. 23. Compared with the TCN-based models, the MAE distribution of the RNN-based models was significantly larger in the new project. In addition, RNN, LSTM, and GRU should be considered inferior to TCN and are therefore not recommended for multistep real-time prediction of the penetration rate. The TCN, when combined with the SENet block, exhibits increased instability and shows a significantly larger error distribution in the 5th step prediction compared to the TCN alone (Supplementary Information).

**Figure 24:**
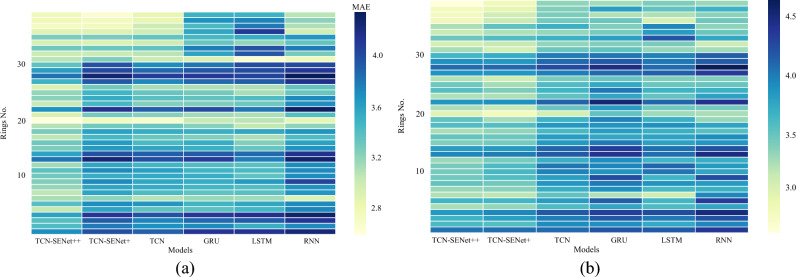
Heatmap of MAE distribution in a new project: (**a**) 5th step prediction, (**b**) 9th step prediction.

In the new project, the MAE of each ring was analyzed, as shown in Fig. 24. In the 5th step prediction, all models except TCN-SENet++ exhibited higher MAE values. TCN-SENet++ demonstrated a significant reduction in prediction errors. In the 9th step prediction, TCN-SENet++ and TCN-SENet+ yielded similar results for rings 1–30. However, for rings 31–40, TCN-SENet++ significantly outperformed TCN-SENet++. The addition of a residual structure based on the SENet block in TCN-SENet++ effectively mitigated the degradation in model performance. Moreover, the rings with high prediction errors correspond to those with a larger standard deviation in the penetration rate (greater than 10). Geological conditions have a substantial impact on TBM construction, leading to considerable variations in penetration rate settings, even under similar underground conditions. A similar conclusion was drawn in that considering longer history lengths poses a challenge for RNN-based models^48^.

**Figure 25:**
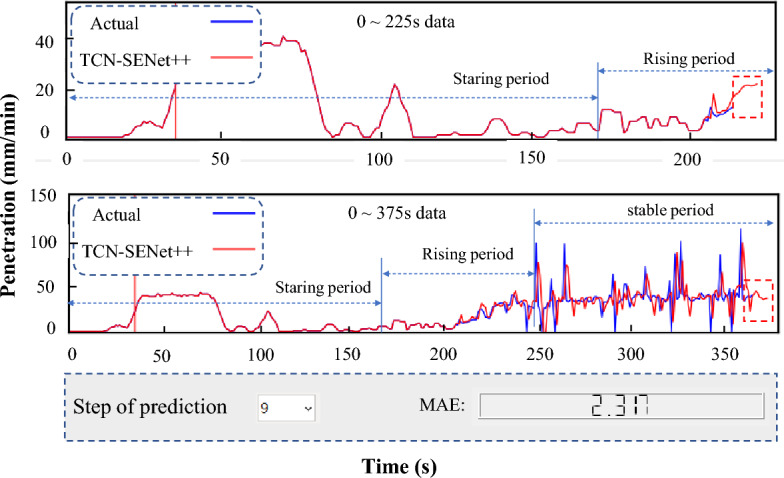
Multi-step real-time penetration rate prediction module.

The TCN-SENet++ model accurately performs dynamic predictions with high performance when historical data on the penetration rates are used as the input. Integrating the developed model into a TBM intelligent construction system enhances the operator's ability to operate a TBM more effectively. TBM tunnel construction is a continuous and dynamic process that involves the collection of construction data. To achieve this, data were collected for each ring for up to 175 s, and the starting and rising periods were divided for a real-time penetration rate prediction. If the rising period was not divided, 5 s were sequentially added to 175 s until the rising period was divided. Figure 25 illustrates the prediction process (9th step prediction) for the first 225 and 375 s of a ring in the penetration rate multistep real-time prediction module. The operator can adjust the prediction time step at any moment based on the real-time MAE to minimize the uncertainty associated with the increasing prediction errors as the prediction step increases.

**Figure 26:**
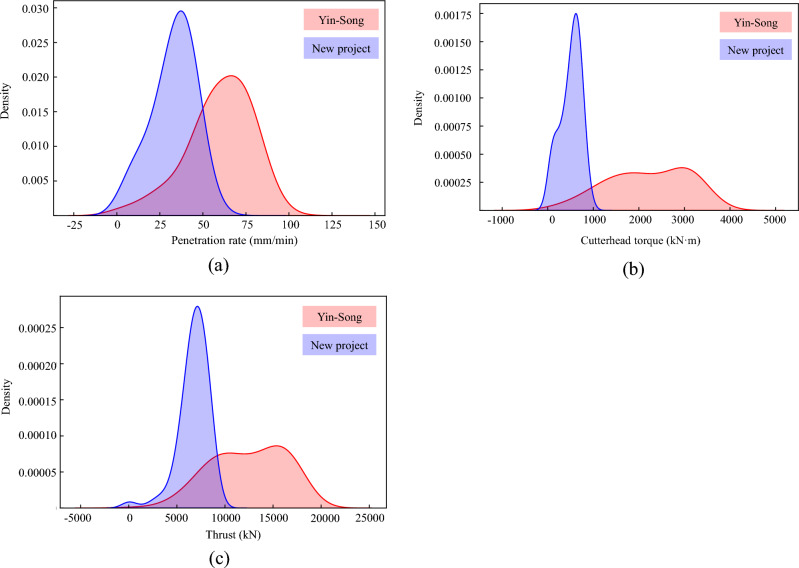
The data distribution of two projects based on kernel density estimation.

The diameter of the TBM was 7.93 m in the Yin-Song diversion project, whereas it was 5.17 m in the new project. This leads to differences in the data distributions for the input features, which require further analysis to address the model transfer considerations. A kernel density estimation was used to compare the datasets from the two projects, as shown in Fig. 26. Both projects exhibit a skewed normal distribution. Increasing the amount of TBM construction data can help align the data to a normal distribution, thereby potentially improving the performance of the penetration rate prediction model. However, the data training process depends on the computer's performance. The surrounding rock grade in the Yin-Song diversion project is Class II, indicating that the Yin-Song data interval used for training encompasses the data interval in the new project, as shown in Fig. 24. During model transfer, the data intervals in the new project should be within those of the developed tunnels.

In “Prediction results of different models” and “Single feature multi-step prediction penetration rate analysis”, the six models were evaluated on the training set, validation set, and test set with multiple features, as well as on the test set with a single feature. The results demonstrated that the performance of TCN-SENet++ was superior to that of RNN, LSTM, GRU, TCN, and TCN-SENet+. The generalization ability of the proposed TCN-SENet++ is evidenced by its performance in the new project. Despite the differences in geological conditions and input feature distributions between the Yin-Song diversion project and the new project, TCN-SENet++ maintained high predictive accuracy. The ability to generalize across different datasets is crucial for practical applications, ensuring that the model can reliably predict penetration rates under diverse conditions. The consistent performance across multiple prediction steps further underscores the model's superior generalization capability. Additionally, the kernel density estimation comparison indicates that while there are differences in data distributions, the model can still perform effectively, highlighting its potential for model transfer. This capability is particularly valuable in engineering applications where geological conditions and other factors can vary significantly between projects. The proposed TCN-SENet++ model's superior performance in both known and new settings confirms its utility and reliability for dynamic and real-time TBM operations.

### Limitation

The proposed model required more training time than the other models owing to the fully connected layers in the SENet block. Currently, there is only a multiphase prediction of the penetration rate based on TBM construction big data, and there is no solution for adjusting the operational parameters under adverse geological conditions (e.g., collapse).

## Conclusion

Accurate prediction of the penetration rate is crucial for optimizing TBM construction and minimizing TBM damage. To address the limitations of the existing models and effectively utilize TBM construction data, we propose a novel hybrid multistep real-time penetration rate prediction model, TCN, fused with SENet. This model improved the connection between historical data and future penetration rates. This was validated using datasets from the Jilin Yin–Song project and a new project, yielding valuable conclusions.

1. The TCN-SENet++ method outperformed other models with multiple features (penetration rate, cutterhead torque, thrust, and cutterhead power) and a single feature (penetration rate), achieving average MSE reductions of 18%, 6%, 3%, 1%, and 2%, respectively. However, the prediction error increased with longer prediction time steps for all the models.
2. The increased TBM vibration during the stable periods caused data dispersion, leading to larger fitting coefficients for the rising period. The rings with significant errors showed a large standard deviation (penetration rate standard deviation greater than 10). Shortening the prediction step is recommended to reduce errors.
3. The average training times for the RNN, LSTM, GRU, TCN, TCN-SENet+, and TCN-SENet++ were 316.718, 518.539, 313.162, 1304.500, 1613.426, and 2353.915s, respectively. The SENet block enhances the relationship between the historical and future penetration rates but increases the computational complexity and training time. Using different time steps affects the multistep real-time prediction of the penetration rate. A 30-s time step reduces the MSE by 4% compared to 15 s, while a 60-s time step increases the MSE by 0.06% compared to 30 s. The improvements are significant up to 33 s but are limited between 33 and 45 s.
4. The skewed normal distribution of the input features in both projects led to increased prediction errors. Applying the model transfer learning ensured that the data intervals of the new project matched those of the developed tunnels. In the new project, the MSE and MAE of the model based on the Yin–Song training were 16.908 and 3.217, respectively. Alternatively, we used the construction data of a new project as the training set to predict the penetration rate of the unconstructed tunnel sections.

In future work, we will actively explore how to achieve a multistep prediction of the penetration rate under adverse geological conditions. In addition, further research is required to assess the applicability of this method to the prediction of other TBM parameters.

### Supplementary Information

Supplementary Information.

## Data Availability

The datasets generated and analyzed during the current study are not publicly available due to the requirements of our partners but are available from the corresponding author upon reasonable request.
