# Supplementary figures and images for: Multi-step real-time prediction of hard-rock TBM penetration rate combining temporal convolutional network and squeeze-and-excitation block
Source: Sci Rep. 2024 Jun 21;14:14326. doi: 10.1038/s41598-024-65351-3 (PMC11192934; doi:10.1038/s41598-024-65351-3)

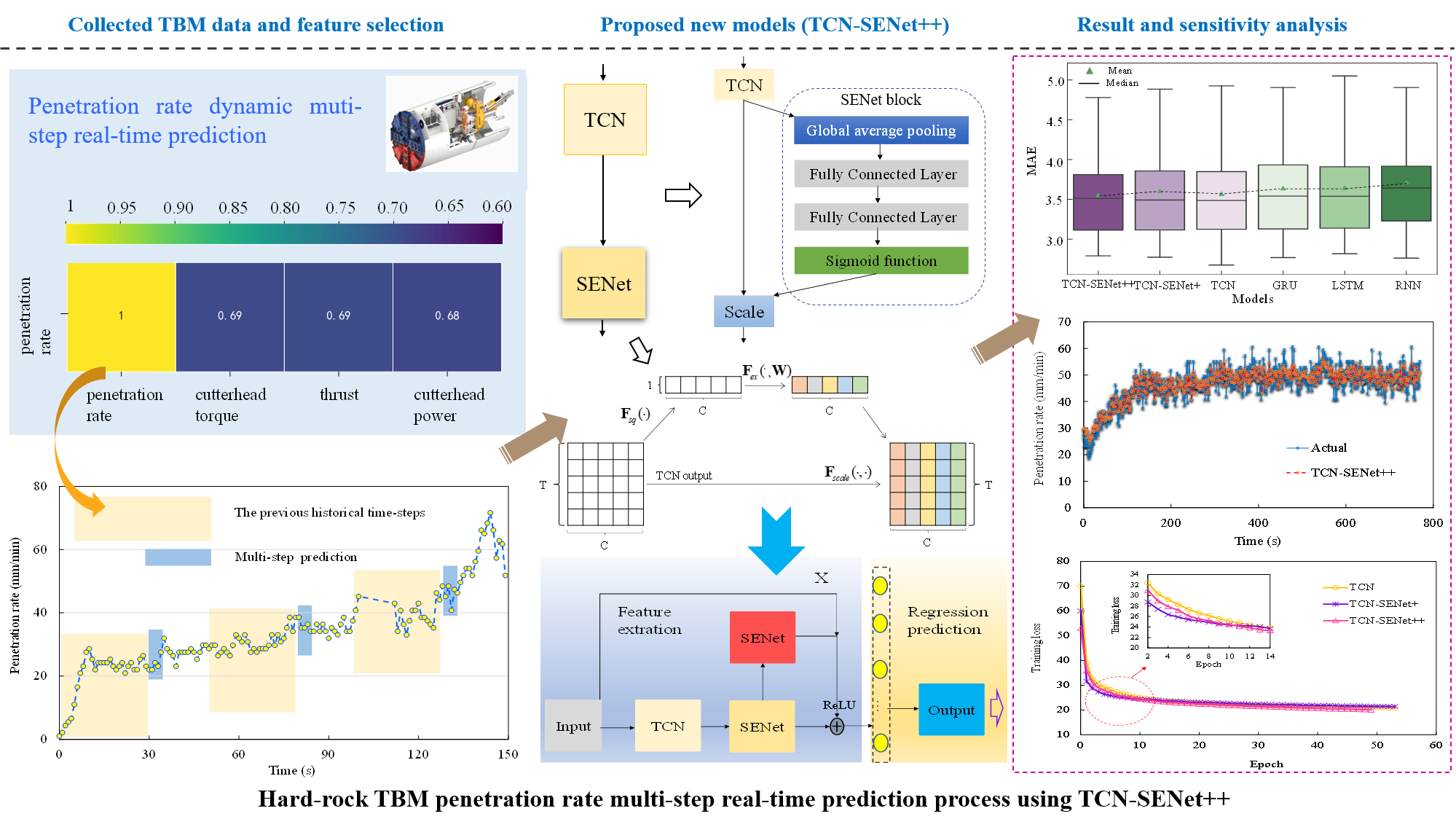

Supplement: Supplementary file 1 — Supplementary Information. [file 41598_2024_65351_MOESM1_ESM.tif]
